# Renal adverse effects following the use of different immune checkpoint inhibitor regimens: A real‐world pharmacoepidemiology study of post‐marketing surveillance data

**DOI:** 10.1002/cam4.3198

**Published:** 2020-07-27

**Authors:** Gang Chen, Yan Qin, Qian‐qian Fan, Bin Zhao, Dan Mei, Xue‐mei Li

**Affiliations:** ^1^ Nephrology Department Peking Union Medical College Hospital Peking Union Medical College Chinese Academy of Medical Sciences Beijing China; ^2^ Pharmacy Department Peking Union Medical College Hospital Peking Union Medical College Chinese Academy of Medical Sciences Beijing China

**Keywords:** Adverse Event Reporting System, immune checkpoint inhibitor, renal adverse effects

## Abstract

**Backgrounds:**

Although kidney impairments have been reported following immune checkpoint inhibitors (ICIs) in clinical studies, there are few pharmacoepidemiology studies to compare the occurrences, clinical features, and prognosis of renal adverse effects.

**Methods:**

Disproportionality and Bayesian analysis were used in data mining to screen the suspected renal adverse effects after the administration of different ICIs, based on FDA's Adverse Event Reporting System (FAERS) from January 2004 to September 2019. The time to onset, fatality and hospitalization rates of renal adverse effects were also investigated.

**Results:**

We identified 1444 reports of renal adverse effects. Affected patients tended to be older than 65 years (52.7%). Renal effects were most commonly reported in nivolumab monotherapy (33.24%). Atezolizumab appeared the strongest association among six ICI monotherapies, based on the highest reporting odds ratio (ROR = 144.38, two‐sided 95% CI = 123.08 −169.37), proportional reporting ratio (PRR = 139.13, *χ* ^2^ = 21 425.38), and empirical Bayes geometric mean (EBGM = 131.75, one‐sided 95% CI = 115.28). The combination treatments showed higher RORs, PRRs, and EBGMs, compared with either nivolumab or pembrolizumab monotherapy. The median onset time of renal adverse effects was 48 (interquartile range [IQR] 18.75‐121.25) days after the monotherapies of ICI regimens. Patients treated with the combination of nivolumab plus ipilimumab were younger than receivers in nivolumab monotherapy (63.81 ± 12.03 vs 66.39 ± 11.53, *P* = .004); The fatality rate of renal adverse effects appeared lower in the combination group, compared to nivolumab monotherapy (18.53% vs 27.50%, *P* = .004). The top hospitalization rates due to renal effects occurred in patients with combination therapies.

**Conclusion:**

Based on the FAERS database, we profiled renal adverse effects after various ICIs with real‐world data in occurrences, clinical characteristics, and prognosis. Renal effects should be tightly monitored, especially within the first several months after ICIs administration. Particular concern should be paid for patients with a tendency for kidney impairments, such as old age.

## Graphical abstract

This is the first and largest pharmacoepidemiology study until recently to compare the associations, timing, and prognosis of renal adverse effects following different immune checkpoint inhibitors (ICIs) in the real‐world practice based on the Food and Drug Administration's Adverse Event Reporting System (FAERS) pharmacovigilance database. Renal adverse effects related to various ICIs are profiled with real‐world data in occurrences, clinical characteristics, and prognosis. We suggest that particular concern should be paid for atezolizumab, nivolumab, and combination therapies when applied for patients with a tendency for renal effects.

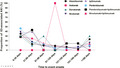

**Abbreviations**
AIN: acute interstitial nephritis
AKI: acute kidney injury
BCPNN: Bayesian confidence propagation neural network
CTLA‐4: cytotoxic T‐lymphocyte‐associated antigen 4
EBGM: empirical Bayes geometric mean
FAERS: the Food and Drug Administration's Adverse Event Reporting System
FDA: the Food and Drug Administration
ICI: immune checkpoint inhibitor
IQR: interquartile range
irAEs: immune‐related adverse events
MGPS: multi‐item gamma Poisson shrinker
PD‐1: programed cell death receptor 1
PD‐L1: programed cell death ligand 1
PRR: proportional reporting ratio
ROR: reporting odds ratio
SRS: spontaneous reporting system

## 1 BACKGROUND

Immune checkpoint inhibitors (ICIs) are effective in approximately 25% of patients with advanced malignancy and have substantially improved the prognosis and revolutionized cancer treatment.^1^ Currently approved ICIs are categorized as anti‐cytotoxic T‐lymphocyte‐associated antigen 4 (CTLA‐4) antibody, ipilimumab; anti‐programed cell death receptor 1 (PD‐1) regimens, including nivolumab and pembrolizumab; and regimens targeting programed cell death ligand 1 (PD‐L1) including atezolizumab, avelumab, and durvalumab (Table 1). Indications for ICI treatment have been expanding for both solid and hematologic malignancies.^2^, ^3^, ^4^, ^5^, ^6^, ^7^

**TABLE 1:** Summary of FDA‐approved ICIs

Generic name	Brand name	Target	Year of approval
Atezolizumab	Tecentriq	PD‐L1	2016
Avelumab	Bavencio	PD‐L1	2017
Durvalumab	Imfinzi	PD‐L1	2017
Nivolumab	Opdivo	PD‐1	2014
Ipilimumab	Yervoy	CTLA‐4	2011
Pembrolizumab	Keytruda	PD‐1	2014

Abbreviations: CTLA‐4, cytotoxic T lymphocyte‐associated antigen 4; FDA, Food and Drug Administration; ICIs, immune checkpoint inhibitors; PD‐1, programed cell death protein 1; PD‐L1, programed cell death ligand 1.

Despite their favorable benefits, the disinhibition of T‐cell function by ICIs can lead to a spectrum of immune‐related adverse events (irAEs), due to the accompanied inflammatory reactions against healthy tissues. Toxicity can affect nearly any organ system, and the common irAEs include dermatitis, colitis, hepatitis, pneumonitis, and thyroid and pituitary impairments.^8^ Renal irAEs have been estimated as rare, with an incidence of about 2% with ICI monotherapy and 5% with combination therapy.^9^ The ICI‐associated renal adverse effects demonstrated a broad spectrum, with acute kidney injury (AKI) most commonly in the form of acute interstitial nephritis (AIN).^8^, ^10^ Although most affected patients respond well to steroids, some severe AKIs induce dialysis‐dependent renal failure or death.^9^, ^11^, ^12^, ^13^

Although there are increasing studies concerning the ICI‐associated renal adverse effects, most evidence origin from cases^12^, ^14^, ^15^, ^16^ or clinical trials,^9^, ^17^ which is still far from enough to understand such a relatively rare adverse event. There is only one pharmacovigilance study generally described ICIs‐mediated reactions in different organs, including the kidney.^18^ Knowledge is scarce about the detailed safety profile of renal adverse effects following various ICIs in real‐world clinical practice. Therefore, we aimed to evaluate and compare the links between different ICIs and renal adverse effects in a large population by investigating the FDA's Adverse Event Reporting System (FAERS) until recently. We further examined the time to onset, fatality rate, and hospitalization rate for renal adverse effects following different ICI regimens.

## 2 MATERIALS AND METHODS

### 2.1 Data source

We performed a retrospective pharmacovigilance study using data from the FAERS database dated from January 2004 to September 2019. The FAERS is a public spontaneous reporting system (SRS) that contains information about adverse drug events provided by health professionals, patients, and manufacturers not only domestically, but also from other regions. FAERS data files describe demographic and administrative information (DEMO), drug information (DRUG), preferred terms (PTs) coded for the adverse events (REAC), patient outcomes (OUTC), report sources (RPSR), therapy start dates and end dates for reported drugs (THER), and indications for drug administration (INDI).

**FIGURE 1:**
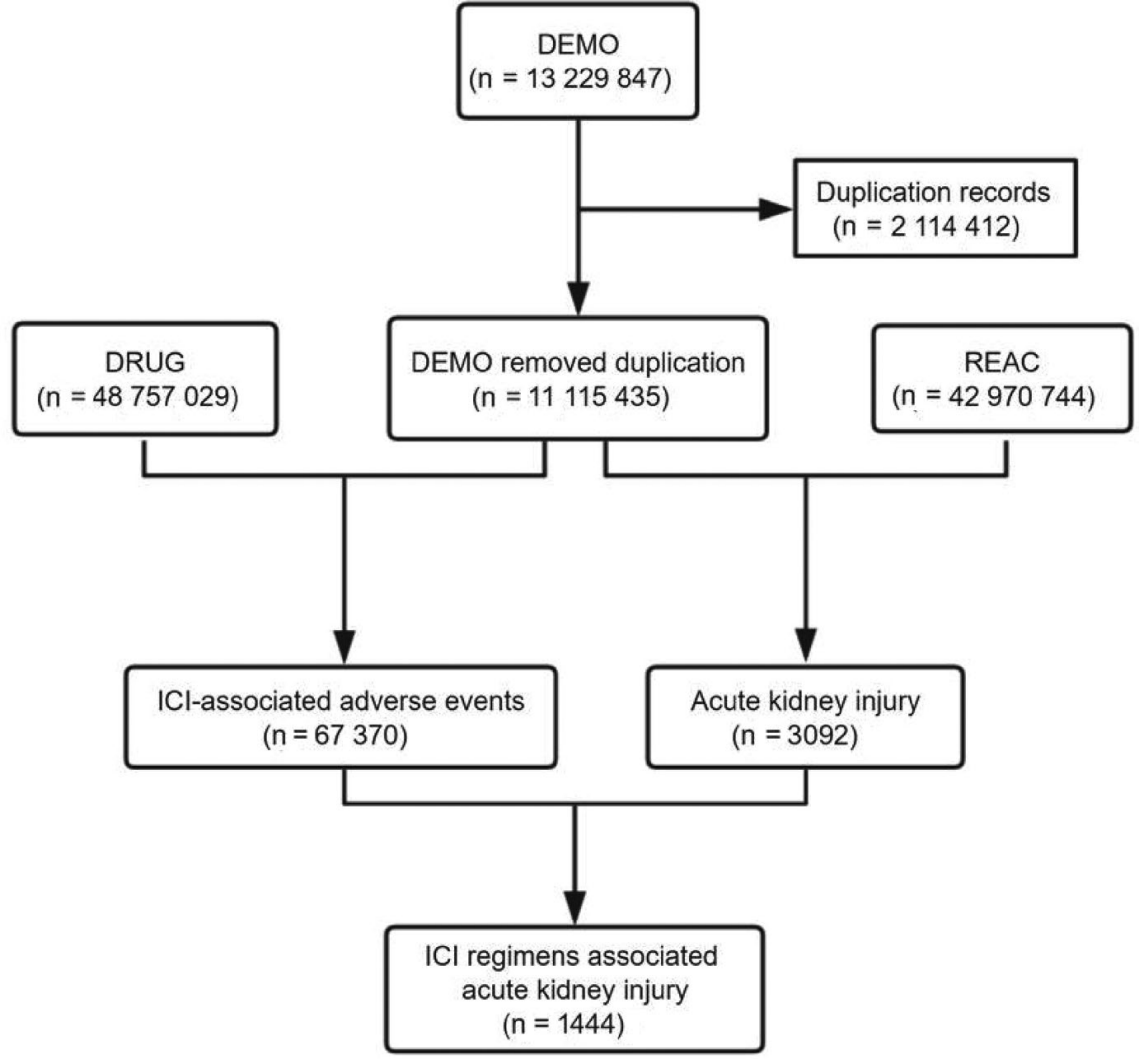
Process of the selection of cases of ICI‐associated renal adverse effects from the FAERS database. Abbreviations: ICI, immune checkpoint inhibitor; FAERS, Food and Drug Administration's Adverse Event Reporting System

We screened 13 229 847 reports from the FAERS database and removed duplicated records according to the FDA's recommendations by selecting the latest FDA_DT when the CASEID and FDA_DT were the same. We finally included 11,115,435 reports for further analysis (Figure 1).

### 2.2 Adverse event and drug identification

We investigated in the REAC files for comprehensive Medical Dictionary for Regulatory Activities (MedDRA), terms related to renal adverse effects as following: “acute kidney injury,” “kidney failure,” “subacute kidney injury,” “oliguria,” “anuria,” “dialysis,” “proteinuria,” “blood creatinine increased,” “blood urea increased,” “nephritis,” “nephropathy toxic,” “tubulointerstitial nephritis,” “renal tubular injury,” “glomerulonephritis acute,” and “glomerulonephritis rapidly progressive.” We chose generic and brand names of ICIs (Table 1) by utilizing the MICROMEDEX (Index Nominum) as a dictionary in the data mining process.

### 2.3 Data mining

Based on the rationale of Bayesian analysis and disproportionality analysis, we employed the reporting odds ratio (ROR), the proportional reporting ratio (PRR), the Bayesian confidence propagation neural network (BCPNN), and the multi‐item gamma Poisson shrinker (MGPS) algorithms to investigate the association between the drug and the given adverse events. We listed the equations and criteria for the four algorithms in Table 2.

**TABLE 2:** Summary of major algorithms used for signal detection

Algorithms	Equation*	Criteria
ROR	ROR = (a/b)/(c/d)	95% CI > 1, N ≥ 2
	95%CI = e^ln(ROR) ± 1.96(1/a + 1/b + 1/c + 1/d)^0.5^	
PRR	PRR = (a/(a + c))/(b/(b + d))	PRR ≥ 2, *χ* ^2^ ≥ 4, N ≥ 3
	*χ* ^2^ = Σ((O‐E)2/E); (O = a, E=(a + b)(a + c)/(a + b+c + d))	
BCPNN	IC = log_2_a(a + b+c + d)/((a + c)(a + b))	IC025 > 0
	IC025 = e^ln(IC) − 1.96(1/a + 1/b + 1/c + 1/d)^0.5^	
MGPS	EBGM = a(a + b+c + d)/((a + c)(a + b))	EBGM05 > 2, N > 0
	EBGM05 = e^ln(EBGM) − 1.64(1/a + 1/b + 1/c + 1/d)^0.5^	

Abbreviations: BCPNN, Bayesian confidence propagation neural network; CI, confidence interval; EBGM, empirical Bayesian geometric mean; EBGM05, the lower 90% one‐sided CI of EBGM; IC, information component; IC025, the lower limit of the 95% two‐sided CI of the IC; MGPS, multi‐item gamma Poisson shrinker; N, the number of co‐occurrences; PRR, proportional reporting ratio; ROR, reporting odds ratio; *χ* ^2^, chi‐squared.

* a: number of reports containing both the suspect drug and the suspect adverse drug reaction. b: number of reports containing the suspect adverse drug reaction with other medications (except the drug of interest). c: number of reports containing the suspect drug with other adverse drug reactions (except the event of interest). d: number of reports containing other medications and other adverse drug reactions.

We compared the associations between renal adverse effects and different ICIs. ICIs can be administrated as monotherapies or combined therapies. Monotherapy meant the application of a specific ICI without other ICI regimens, and this specific ICI was identified as “primary suspect” in the ROLE_COD field of DRUG files without other ICIs being listed as “second suspect,” “concomitant,” or “interacting.” Combined therapy meant the concurrent usage of PD‐1, PD‐lL, or CTLA‐4 inhibitors. In this scenario, a specific ICI was notified as “primary suspect,” with another ICI being listed as “second suspect,” “concomitant,” or “interacting.” We also evaluated the onset time to renal adverse effects for different ICIs, which was defined as the interval between the EVENT_DT (adverse event onset date) and the START_DT (start date of the ICIs administration). We excluded the records with incorrect entry or erred input (EVETN_DT earlier than START_DT). Additionally, we analyzed reports with fatal events due to adverse drug reactions and calculated the fatality rate as dividing the fatal events by the total number of ICI‐associated renal adverse effects.

### 2.4 Statistical analysis

We used descriptive analysis to summarize the clinical features of the patients with ICI‐associated renal adverse effects from the FAERS database. The onset times to renal adverse effects among different ICIs were compared using nonparametric tests (the Mann‐Whitney test for dichotomous variables and the Kruskal‐Wallis test when there were more than two subgroups of respondents). Pearson's chi‐square test or Fisher exact test was used to comparing the fatality rates between different ICIs. The statistical significance was determined at *P* < .05 with 95% confidence intervals. Data mining and the statistical analysis were performed by SAS, version 9.4 (SAS Institute Inc.).

## 3 RESULTS

### 3.1 Descriptive analysis

About 67 370 adverse events related to ICIs and 3092 reports related to renal adverse effects were documented in the FAERS database dated from January 2004 to September 2019 (Figure 1). We have screened 1444 reports with suspected ICI‐related renal adverse effects and summarized the clinical features of these patients in Table 3. Most cases were reported from North America (39.82%) and Europe (36.15%) and were submitted by health‐care professionals (81.44%). The reported cases of renal adverse effects have gradually increased from 2011 to 2019. Affected patients tended to be older than 65 years (52.7%) and were often more male than female (62.12% vs 32.83%), with an average age of 66.22 ± 10.79 years for male and 65.47 ± 12.59 years for female. Patients administrated with combination therapies of nivolumab plus ipilimumab tended to be younger than receivers in nivolumab monotherapy (63.81 ± 12.03 vs 66.39 ± 11.53, *P* = .004). Nivolumab monotherapy generated the largest number of reports associated with renal adverse effects (n = 480, 33.24%) in our study, followed by combination therapy of nivolumab plus ipilimumab (n = 340, 23.55%). Patients with thorax cancer reported the most substantial amount of renal adverse effects in the FAERS database (32.13%), followed by skin cancer (29.02%) and genitourinary cancer (17.17%).

**TABLE 3:** Clinical characteristics of patients with ICI‐associated renal adverse effects sourced from the FAERS database (January 2004 to September 2019)

Characteristics	Reports, no. (%)
Reporting region	
Europe	522 (36.15%)
North America	575 (39.82%)
South America	32 (2.22%)
Asia	261 (18.07%)
Oceania	51 (3.53%)
Africa	2 (0.14%)
Unspecified	1 (0.07%)
Reporters	
Health‐care professionals	1176 (81.44)
Non‐health‐care professional	267 (18.49)
Reporting year	
2019	471 (32.62)
2018	429 (29.71)
2017	239 (16.55)
2016	171 (11.84)
2015	104 (7.20)
2014	16 (1.11)
2013	7 (0.48)
2012	5 (0.35)
2011	2 (0.14)
Sex of patients	
Male	897 (62.12)
Female	474 (32.83)
Unknown or missing	73 (5.06)
Age groups (years)	
<18	2 (0.14)
18‐44	53 (3.67)
45‐64	428 (29.64)
65‐74	519 (35.94)
>75	242 (16.67)
Unknown or missing	200 (13.85)
ICI drugs as suspected drugs	
Monotherapy	
Atezolizumab	165 (11.43)
Avelumab	3 (0.21)
Durvalumab	13 (0.90)
Nivolumab	480 (33.24)
Ipilimumab	103 (7.13)
Pembrolizumab	325 (22.51)
Combination therapy	
Pembrolizumab + Ipilimumab	15 (1.04)
Nivolumab + Ipilimumab	340 (23.55)
Indications for tumors of different sites	
Head and neck	18 (1.25)
Digestive system	79 (5.47)
Thorax	464 (32.13)
Musculoskeletal sites	9 (0.62)
Skin	419 (29.02)
Breast	13 (0.90)
Gynecologic sites	28 (1.94)
Genitourinary sites	248 (17.17)
Ophthalmic sites	8 (0.55)
Central nervous system	1 (0.07)
Hematopoietic and lymphoid tissues	36 (2.49)
Unspecified or missing	121 (8.38)

Abbreviations: FAERS, Food and Drug Administration's Adverse Event Reporting System; ICI, immune checkpoint inhibitor.

### 3.2 Disproportionality analysis and Bayesian analysis

We detected signals of renal adverse effects for all 6 ICI monotherapies and 2 ICI combination therapies based on the criteria for the four algorithms and listed the results in Table 4. Among all ICI monotherapies, atezolizumab was particularly noteworthy for the relationship to renal adverse effects due to its highest ROR, PRR, and EBGM; pembrolizumab ranked the second; whereas avelumab appeared to show a relatively weaker association with renal adverse effects than others. For the therapies of dual regimens, the combined ICIs of ipilimumab plus nivolumab or pembrolizumab appeared stronger associations with renal adverse effects than the single regimen of either nivolumab or pembrolizumab, based on the higher RORs, PRRs, and EBGMs. No other combined regimens of anti‐CTLA‐4 plus anti‐PD‐1/PD‐L1 have been reported in the FAERs database.

**TABLE 4:** Association of different ICI regimens with renal adverse effects

Drug	N	ROR (95% two‐sided CI)	PRR (*χ* ^2^)	IC (IC025)	EBGM (EBGM05)
Atezolizumab	165	144.38 (123.08, 169.37)	139.13 (21 425.38)	7.04 (6)	131.75 (115.28)
Avelumab	3	20.75 (6.67, 64.59)	20.64 (56.03)	4.37 (1.4)	20.62 (7.97)
Durvalumab	13	33.68 (19.48, 58.21)	33.37 (406.66)	5.05 (2.92)	33.24 (21.03)
Nivolumab	480	74.74 (67.76, 82.44)	73.47 (28 991.64)	5.96 (5.4)	62.22 (57.31)
Ipilimumab	103	42.27 (34.69, 51.5)	41.8 (3966.76)	5.34 (4.38)	40.45 (34.28)
Pembrolizumab	325	88.84 (79.11, 99.78)	86.94 (24 713.54)	6.28 (5.6)	77.91 (70.7)
Pembrolizumab + Ipilimumab	15	176.45 (104.96, 296.64)	168.28 (2482.82)	7.39 (4.39)	167.46 (108.43)
Nivolumab + Ipilimumab	340	163.71 (145.97, 183.6)	157.36 (47 029.57)	7.13 (6.36)	140.17 (127.34)

Abbreviations: CI, confidence interval; EBGM, empirical Bayes geometric mean; IC, information component; ICI, immune checkpoint inhibitor; N, the number of reports of ICI‐associated renal adverse effects; PRR, proportional reporting ratio; ROR, reporting odds ratio; *χ* ^2^,: chi‐squared.

### 3.3 Time to onset of ICI‐associated renal adverse effects

**FIGURE 2:**
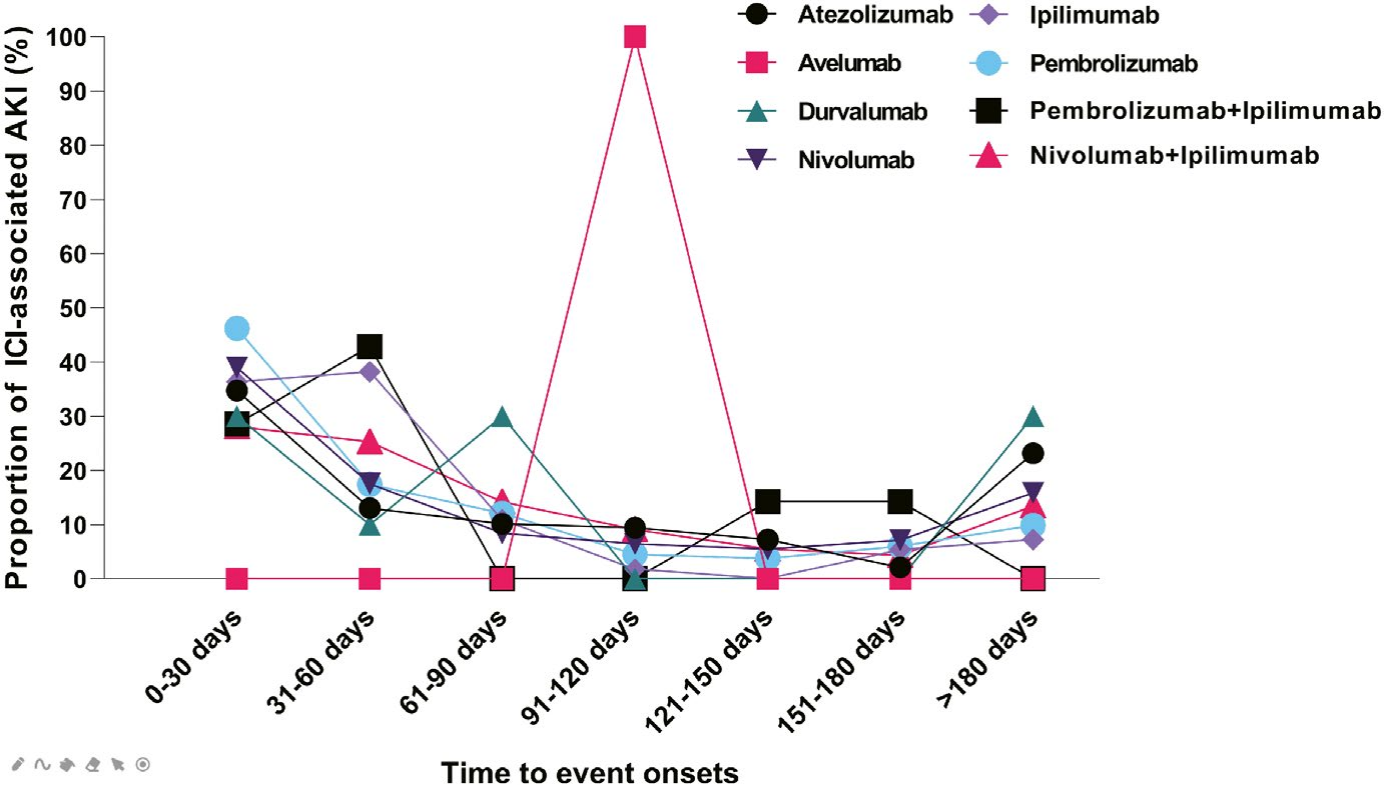
Time to event onset of renal adverse effects following immune checkpoint inhibitor regimens

Overall, the median onset time to renal adverse effects was 48 (interquartile range [IQR] 18.75‐121.25) days. We described the onset time for each ICI regimen in Figure 2. Noteworthily, renal adverse effects could occur as soon as the first dose after atezolizumab, nivolumab, and pembrolizumab, indicated by the identical dates of drug administration and adverse event onset in the database. The median times to renal adverse effects onset among different ICI monotherapies were 38 (IQR 26.75‐60) days for ipilimumab, 40 (IQR 10‐86) days for pembrolizumab, 51 (IQR 14‐139) days for nivolumab, 63 (IQR 20.5‐167.5) days for atezolizumab, and 66 (IQR 11‐146.5) days for durvalumab, respectively. We identified a significant difference in time to renal adverse effects among all ICI regimens (Kruskal‐Wallis test, *P* = .038). We found no difference in onset times to renal adverse effects between patients treated with nivolumab monotherapy and the combination of nivolumab plus ipilimumab (Mann‐Whitney test, *P* = .231), nor between patients with single pembrolizumab and dual regimens of pembrolizumab plus ipilimumab (Mann‐Whitney test, *P* = .306). The median onset time to renal adverse effects for nivolumab plus ipilimumab was 55.5 (IQR 27‐119.25) days, compared with 51 (IQR 14‐139) days for nivolumab monotherapy.

### 3.4 Fatality and hospitalization due to ICI‐associated renal adverse effects

**FIGURE 3:**
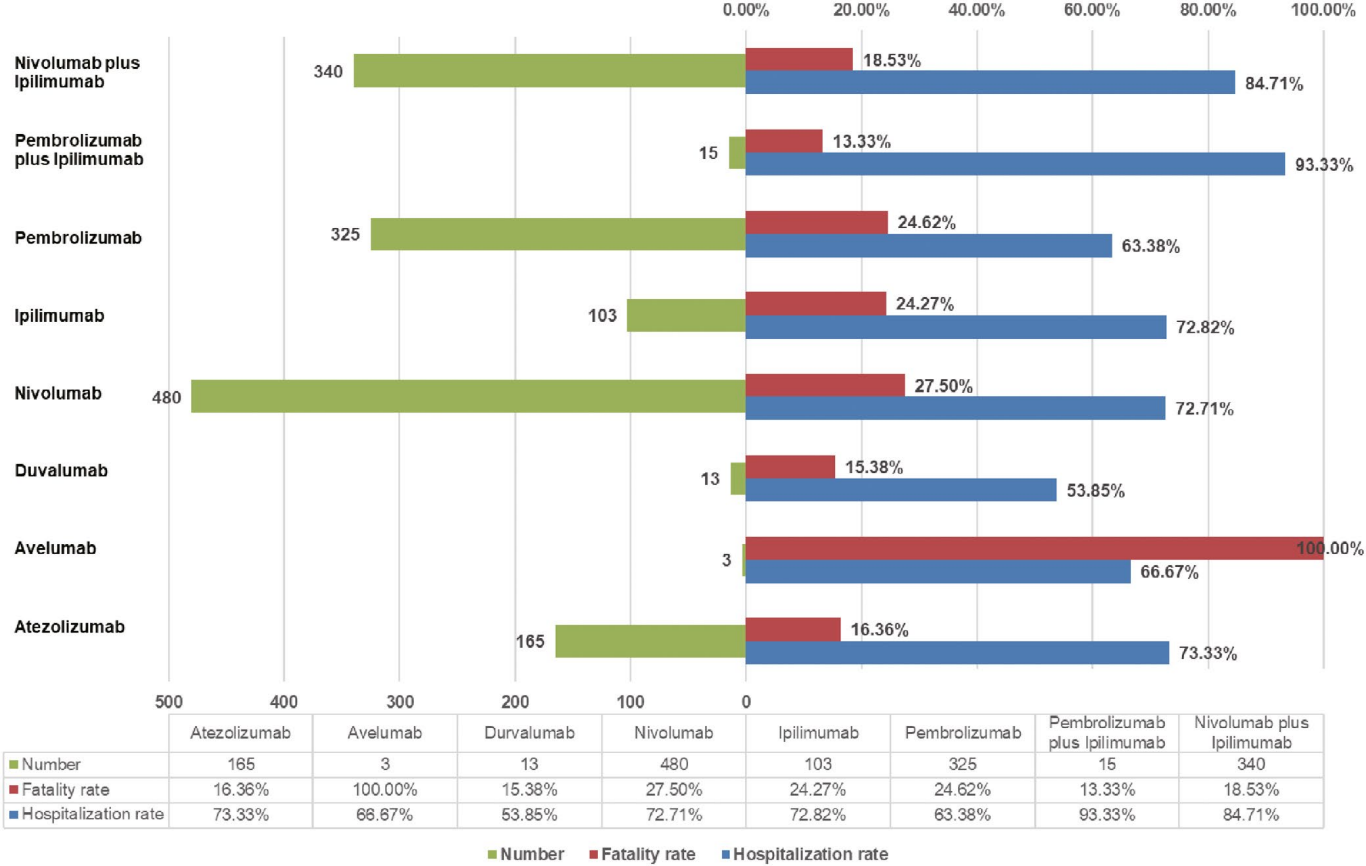
The number of reports, hospitalization rates, and fatality rates for ICI‐associated renal adverse effects. ICI indicates an immune checkpoint inhibitor

To analyze the prognosis of ICI‐associated renal adverse effects, we assessed the rates of fatality and hospitalization due to renal effects following ICI treatments and generated the results in Figure 3. In our analysis, we found the outcome of renal adverse effects tended to be poor, generally resulting in 73.55% hospitalization and 23.13% death. Among monotherapies, fatal events have alarmingly happened in three out of three (100%) cases of avelumab treatment. Other than avelumab, there was no significant difference in fatality rates across different ICI monotherapies (Fisher exact test for overall comparison, *P* = .06). The therapy of nivolumab plus ipilimumab resulted in a lower death rate than nivolumab monotherapy (18.53% vs 27.50%, *P* = .004). There was no significant difference among the death rates for pembrolizumab, ipilimumab, and the combination of both. We did not find a significant difference in death rates between the dual regimens of nivolumab plus ipilimumab and the combination of pembrolizumab and ipilimumab (18.53% vs 13.33%, *P* = .748). The top two hospitalization rates due to renal adverse effects occurred in the two combination therapies. The hospitalization rate in nivolumab plus ipilimumab was significantly higher than that in nivolumab monotherapy (84.71% vs 72.71%, *P* < .001). Similarly, the hospitalization rate in pembrolizumab plus ipilimumab was significantly higher than that in pembrolizumab monotherapy (93.33% vs 63.38%, *P* = .024).

## 4 DISCUSSION

To the best of our knowledge, this study is the first and largest collection until recently to compare the associations, timing, and prognosis of renal adverse effects after different ICIs in the real‐world practice based on the FAERS pharmacovigilance database. All the 6 ICIs in this study demonstrated association with renal adverse effects and presented diverse characteristics.

ICI‐associated renal adverse effects have attracted clinical attention (10, 14), but the assessment and characterization of which are challenging due to their low incidence, various manifestations, and limited post‐marketing time of novel drugs. Ipilimumab is the first FDA‐approved ICI, and renal effects have been noted in its early trials in melanoma, among which acute interstitial nephritis (AIN) has mostly been reported.^9^, ^17^ A spectrum of glomerular lesions has also been mentioned in ipilimumab‐induced kidney diseases.^15^, ^19^ Similarly, in the trials of pembrolizumab and nivolumab, evidence alarmed renal adverse effects in forms of AIN and nephritis.^2^, ^20^, ^21^, ^22^, ^23^, ^24^ Severe renal adverse effects have not been notable in trials of other three newer PD‐1/PD‐L1 inhibitors,^5^, ^25^, ^26^, ^27^, ^28^, ^29^, ^30^, ^31^, ^32^, ^33^, ^34^ but symptoms indicating renal lesion, such as proteinuria, edema, and moderate creatinine increase also appeared in treatments with avelumab,^32^, ^33^ atezolizumab^30^, ^31^ and durvalumab.^29^, ^34^

Tremendous can be learned from clinical trials since they are mandatory to investigate the adverse effects of novel treatments. However, they still lack enough power to draw definitive conclusions about drug safety due to the strict inclusion criteria, limited sample sizes, and relatively short observation periods. Importantly, SRS can serve as a primary source of post‐marketing data leading to ICIs safety issues, including renal adverse effects.^35^ In our study based on the FAERS, we noticed the rapidly incremental reports year by year, which may be explained by the widely expanding indications of ICIs and intensively spreading clinical practice. ICI‐associated renal adverse effects appeared to affect more men (62.12%) than women in our data, which was also mentioned in another summary.^19^ However, remarkably there was a more robust representation of male patients in a series of ICIs trials,^36^, ^37^ which was doubted as some higher mutation rates in women with tumors that might influence ICIs application.^38^ Therefore, sex disparity in renal adverse effects needs further well‐designed studies to describe. Our study also indicated that ICI‐mediated renal adverse effects were dominated in elderly generations (52.7% ≥65years) since advanced age was a risk factor for both various cancers^39^ and declined kidney function.^40^ Our data suggest that we should be aware of the renal function among elderly patients who received ICI regimens.

In the pharmacovigilance investigation, we found all ICIs were relevant to renal adverse effects. To our surprise, atezolizumab appeared to have the strongest association among all ICI monotherapies, which has not been alarmed in many previous trials.^5^, ^25^, ^27^, ^30^, ^31^ Two reasons may explain this. First, atezolizumab is a more recently developed ICI, and time is needed to recognize the inconspicuous renal effects with a relatively low incidence and various forms. The progress of gradual understanding of the renal events in nivolumab and pembrolizumab are suitable examples. The FDA approved pembrolizumab and nivolumab in 2014. In the early stage trials of both regimens, they were not considered to have side‐effects in the kidney.^24^, ^41^, ^42^ Now, it is appreciated that renal adverse effects do occur in them. Recent pembrolizumab trials have reported 6.7% of renal adverse effects,^20^, ^21^ while nivolumab trials have reported 3%‐5%.^22^, ^23^, ^24^ These previous observations indicate that the more accurate description of atezolizumab‐associated renal adverse effects is still on its way. Second, the currently available data may underestimate the real incidence due to misdiagnoses or under‐reporting.^14^ Take AKI for the representative of renal adverse effects. Severe AKI is easier to identify, but a smaller rise in creatinine may be neglected or be contributed to various causes such as other suspicious drugs. In some recent studies, AKI is reported as high as 17% in patients following ICIs within 12 months, based on the detection of 1.5 times rise of baseline creatinine.^43^ Besides, we found the concurrent administration of ipilimumab with either nivolumab or pembrolizumab indicated a stronger association with renal adverse effects compared with monotherapies. Although dual ICIs have shown a higher risk for the kidney,^9^ such investigations still need further verification by well‐designed clinical trials of single or dual ICIs to balance the benefits and risks of combined regimens.

Another chief finding was that the median onset time to renal adverse effects was 48 (IQR 18.75‐121.25) days after monotherapies of ICI regimens, which was much earlier than previous observation in a cohort with the median time to onset of 91 (IQR 60‐183) days.^9^ Consciousness is also suggested for the phenomena of immediate occurrence of renal adverse effects after the first‐dose administration of atezolizumab, nivolumab, and pembrolizumab. Judging from the average onset time, it seems that ipilimumab, as an anti‐CTLA4 compound, leads to renal adverse effects in a shorter time than other anti‐PD1/PD‐L1 compounds. This finding is relevant to the previous description of renal adverse effects occurring in 6‐12 weeks after ipilimumab.^19^ The wide‐ranged onset time distribution indicates the varied period to develop renal adverse effects posttreatments, which could be up to several months or even a year after the end of the treatment.^12^ The dual regimens of nivolumab and ipilimumab do not seem to shift the onset of renal adverse effects earlier, but there is no enough data to draw a solid conclusion for dual regimens with pembrolizumab plus ipilimumab (n = 15).

To further compare the severity of ICI‐associated renal adverse effects, we investigated the fatality and hospitalization rates. Renal adverse effects generally led to a concerning outcome with a 23.13% fatality rate. Avelumab resulted in three death out of three patients, but the data were too scarce to picture its exact fatality rate. Regarding the death rates of other monotherapies, there was no significant difference, although the nivolumab ranked the highest of 27.5%. Surprisingly, the death rate of single nivolumab was higher than its combination with ipilimumab. Such finding in a real‐world study seems a controversy with some other trials.^44^ The previous research indicated renal adverse effects with a slightly higher rate and more frequent severity in combination therapies; however, the death induced by renal adverse effects was not mentioned.^44^ Several reasons may account for this controversy. First, the data from the real‐world is different from that of clinical trials. Within trials, strict patient selection criteria and concrete study frames are required, with consideration of balancing the sex, age, and comorbidities. However, in real‐world clinical practice, dual regimens are presumably administrated in younger patients with fewer comorbidities. As evidenced in our study, patients in the group of nivolumab plus ipilimumab were significantly younger than those treated with nivolumab monotherapy (*P* = .004). The benefit at a younger age may overcome the possible adverse effects induced by dual regimens. Second, the indications of combination therapies are more selected in contrast to those of anti‐PD‐1 monotherapies, and the doses in compositions of the combination are usually reduced compared to typical treatment in monotherapies.^3^, ^44^, ^45^, ^46^ Third, the hospitalization due to renal adverse effects occurred more frequently in combination therapies than in nivolumab monotherapy (*P* < .001). This finding indicates the possibility in combination groups of the more organized monitoring for renal function and more intense care after the occurring of renal adverse effects.

Nowadays, the application of ICIs is increasingly penetrating in daily oncology practice. Clinicians should be alerted for renal adverse effects across various ICI regimens. The present findings can be applied in clinical decisions over the choice of ICI treatments and the organization of further monitoring, in light of patient ages, basal renal function, and tendencies of different ICIs on mediating kidney impairments.

Despite the advantages of real‐world research and the data mining techniques in this study, we admit that some particular analysis of adverse drug reaction signals is not feasible based on the SRS. Therefore, there are some limitations to this study. First, voluntary reporting in the database could not cover all ICI‐associated renal adverse effects that happened in the real‐world. Second, during the process of data mining, we noticed the imperfection of information, such as incorrect inputs and incomplete reports, which may lead to bias in the analysis. Third, the data available in SRS only cover patients with adverse effects. Some relevant statistics, such as the incidence rate for each suspicious drug, cannot be calculated due to the lack of total numbers of patients receiving treatment. Besides, it is challenging to identify risk factors between ICIs and renal adverse effects, since the deficiency of preexisting renal diseases and comorbidities that may have impacts on renal function. Although there is some inherited limitation in the FAERS database, it signals some critical aspects of ICI‐associated renal adverse effects, providing clues for further well‐designed researches.

## 5 CONCLUSION

In the present study, we identified signals for renal adverse effects following various ICIs in real‐world practice based on the FAERS database. One distinct finding surfaced from this study is that atezolizumab shows a relatively stronger association with renal adverse effects than other ICIs, while combined ICI regimens strengthen their association with renal adverse effects than monotherapies. Moreover, we should monitor renal effects tightly, especially within the first several months after ICIs administration, and awareness should be raised for some immediate renal adverse effects induced by the first dose of atezolizumab, nivolumab, and pembrolizumab. Besides, old age may be a more significant risk factor than different ICI identities in the association of renal adverse effects. Our findings pave the way for continued pharmacovigilance investigation, and further studies are encouraged to test the hypotheses generated in this study.

## AUTHORS' CONTRIBUTIONS

GC designed the study, analyzed and interpreted data, generated figures and tables, as well as wrote the manuscript draft. BZ designed the study and directed the data mining in the FAERS database. YQ, QQF, DM, and XL reviewed and corrected the manuscript.

## ETHICS APPROVAL AND CONSENT TO PARTICIPATE

Not applicable.

## CONSENT FOR PUBLICATION

Not applicable.

## COMPETING INTEREST

The authors declare that they have no competing interests.

## Data Availability

All necessary data have been presented as tables and figures in the manuscript. Related information is accessible under request to the corresponding author.
